# Prenatal Zinc Deficient Mice as a Model for Autism Spectrum Disorders

**DOI:** 10.3390/ijms23116082

**Published:** 2022-05-29

**Authors:** Ann Katrin Sauer, Simone Hagmeyer, Andreas M. Grabrucker

**Affiliations:** 1 Department of Biological Sciences, University of Limerick, V94 T9PX Limerick, Ireland; ann.katrin.sauer@ul.ie (A.K.S.); simone.hagmeyer@gmail.com (S.H.); 2 Bernal Institute, University of Limerick, V94 T9PX Limerick, Ireland; 3 Health Research Institute (HRI), University of Limerick, V94 T9PX Limerick, Ireland

**Keywords:** autism, mouse models, trace metals, ASD, biometals, Shank3, synaptopathy, copper, MIA

## Abstract

Epidemiological studies have shown a clear association between early life zinc deficiency and Autism Spectrum Disorders (ASD). In line with this, mouse models have revealed prenatal zinc deficiency as a profound risk factor for neurobiological and behavioral abnormalities in the offspring reminiscent of ASD behavior. From these studies, a complex pathology emerges, with alterations in the gastrointestinal and immune system and synaptic signaling in the brain, as a major consequence of prenatal zinc deficiency. The features represent a critical link in a causal chain that leads to various neuronal dysfunctions and behavioral phenotypes observed in prenatal zinc deficient (PZD) mice and probably other mouse models for ASD. Given that the complete phenotype of PZD mice may be key to understanding how non-genetic factors can modify the clinical features and severity of autistic patients and explain the observed heterogeneity, here, we summarize published data on PZD mice. We critically review the emerging evidence that prenatal zinc deficiency is at the core of several environmental risk factors associated with ASD, being mechanistically linked to ASD-associated genetic factors. In addition, we highlight future directions and outstanding questions, including potential symptomatic, disease-modifying, and preventive treatment strategies.

## 1. Introduction

Autism spectrum disorders (ASD) are neurodevelopmental disorders characterized by delayed speech acquisition, deficits in social interactions, and stereotypical behaviors. Genetic studies using large cohorts of patients have shown that the pathogenesis of ASD has a strong genetic component [1], and pathogenic mutations were identified in genes coding for proteins that are often found localized to synapses in the Central Nervous System (CNS) [2,3,4]. For example, from mutations identified in adhesion proteins of the Neuroligin and Neurexin families [5], scaffold proteins of the postsynaptic density (PSD) such as members of the SHANK (SH3 and multiple ankyrin repeat domains) family) [6,7,8], and mutations in mTOR (mechanistic target of rapamycin) signaling [9], a model emerged by which synaptic proteins with identified ASD-associated mutations disrupt a common signaling pathway in ASD.

However, despite identifying this synaptic pathway, the considerable heterogeneity of identified candidate genes that fall outside this model imposes a significant challenge to define a possible common theme. More importantly, ASD also have a strong environmental component. The risk-associated genetic variants are rare in the ASD population, meaning that no single genetic factor has accounted for much more than 1% of all cases of ASD. Furthermore, combining all inherited common variants that each contributes only very low risk, inherited rare variants, and de novo variants (CNVs and SNVs), only ~52% of ASD cases can be explained [2]. Therefore, alternative or additive and modifying causes [10] have to be considered to explain the causes and heterogeneity of ASD.

The perinatal period, comprising intrauterine and early postnatal phases, is a critical time during which exposure to different environmental factors may lead to altered neurobiological development. Several studies have provided links between perinatal zinc deficiency and the development of ASD in humans. For example, a significantly increased incidence of zinc deficiency in autistic patients compared to controls [11,12,13,14,15,16] was found and supported by several meta-analysis studies [17,18,19,20]. Further, an elevation of copper in samples of subjects with autism [21] was reported, and the Cu/Zn ratio found increased in subjects with autism [22,23]. Based on recent data, zinc deficiency, especially maternal zinc deficiency during pregnancy, may be a risk factor for ASD in humans [15,24].

A causal link between prenatal zinc deficiency and ASD, including neuropsychological symptoms, learning and memory impairments [25] and behavioral and emotional problems, has been confirmed in animal models [26]. Moreover, zinc deficiency has been associated with the occurrence of seizures [27] that many individuals with ASD display as a comorbidity [28,29].

Zinc is highly concentrated in the brain, acting as a neurotransmitter/neuromodulator and signaling ion [30,31]. It localizes into presynaptic glutamate-containing vesicles at excitatory “zincergic” synapses [32] but also localizes to the postsynaptic scaffold within these synapses [33,34]. During early brain development, specific patterns of zincergic innervation emerge in the brain [35]. This zincergic innervation seems to serve some early pioneer function, laying down a substrate for later development, and may be involved in establishing hemisphere lateralization [36].

In the adolescent and adult brain, zincergic neurons are part of a subnetwork of projections found within the cortical system. Zincergic projections seem to mainly link cortical and limbic structures [37]. A plethora of zinc transporters and zinc buffering proteins regulates zinc homeostasis in the brain. Two families of zinc transporters: Zrt and Irt-like protein (ZIP), or Solute Carrier Family 39 (SLC39), and the zinc transporters ZnT, also known as SLC30, have been identified [38]. ZIP transporters increase intracellular zinc levels by promoting zinc uptake from the extracellular environment or by mediating the release of zinc from intracellular stores. In contrast, ZnT transporters mediate the efflux of zinc from cells into the extracellular fluid or sequester zinc into intracellular vesicles. ZnT3, for example, is the zinc transporter responsible for loading zinc into presynaptic vesicles [39,40,41]. Interestingly, ASD-like behavioral features were reported in *ZnT3* knockout (KO) mice [42,43]. Further, proteins involved in the regulation of zinc levels such as ZnT5 [44,45], metallothioneins (MTs) such as the brain-specific MT-3 [46], and S100B [47] are ASD candidate genes [48,49,50].

Several studies using prenatal zinc deficiency to understand the causative mechanisms underlying the ASD-like behavior seen in prenatal zinc deficient (PZD) mice have been published in recent years. These studies revealed a complicated interplay between synaptic pathology and gastrointestinal and immune alterations, such as inflammation.

## 2. Prenatal Zinc-Deficient Mice

In contrast to genetic factors, whose effects are often dependent on gene dosage with 50% of the gene expressed in heterozygous animals and an absence of expression in homozygous knockout animals, zinc deficiency occurs across a broad spectrum of possible concentrations. Severe maternal zinc deficiency that can be generated by diets containing less than 1 mg Zn/kg/day was shown to have teratogenic effects and results in gross anatomical abnormalities in the offspring [51,52]. In contrast, mild maternal zinc deficiency throughout pregnancy, which may closely resemble most cases of (subclinical) zinc deficiency in humans, does not result in gross anatomical malformations in the offspring. Besides, no significant differences regarding their general health, motor coordination, muscle tone, and neurological reflexes were observed [53].

Despite a significant reduction in blood zinc levels of pregnant mothers on a mildly zinc-deficient diet, the behavior of mothers was not affected by the treatment regarding ASD-related behaviors [54]. In contrast, although the offspring of these animals received milk with adequate zinc levels after birth through foster mothers and had no signs of acute zinc deficiency at the time of behavioral assessments, the offspring of mothers suffering from mild zinc deficiency during pregnancy showed molecular, brain morphological and behavioral alterations as adults reminiscent of ASD [36,53,54].

### 2.1. Behavioral Impairments of PZD Mice

To confirm a causal relationship between perinatal zinc deficiency and ASD, a detailed behavioral characterization of PZD animals regarding a possible ASD-like phenotype was performed [36,53,54]. Several test paradigms to evaluate the three core symptoms associated with ASD: aberrant reciprocal social interactions, repetitive behavior, as well as impairments in communication were conducted [55]. Moreover, the presence of features resembling co-morbidities often observed in human patients, such as increased anxiety and mental retardation, were assessed.

Cross fostering of the offspring restored brain zinc levels in prenatal zinc-deficient mice as early as postnatal day 3. Therefore, prenatal zinc deficiency targeted a critical time window of prenatal and early postnatal brain development, a significant period of synaptogenesis within the CNS [56,57,58].

A hallmark of ASD symptomatology is the impairment in communication [59,60]. PZD mice show a significant increase in the latency to call and a decrease in the number of calls, sound pressure level, as well as the percentage of calls with overtones and harmonics [54]. The majority of these parameters has also been reported to be affected in several genetic ASD mouse models, for example, *Shank1*, *Shank2*, and *Shank3* knockout mice [61,62,63,64,65,66,67] (Table 1).

Prenatal zinc deficiency also affects social-related traits. Altered social behavior has been reported in multiple studies on PZD animal models [26]; however, prenatal zinc deficiency seems to affect specific traits of sociability. For example, already in 1978, it was reported that prenatal zinc deprived male rats displayed increased aggression and reduced affiliation towards conspecifics [68], and further studies reported altered social emotionality in PZD rats [69,70]. In a three-chamber test, male PZD mice spent more time in the chamber containing a stranger mouse and displayed increased sniffing time toward the provided stranger mouse. This altered social approach behavior has also been described in the Neurexin1 alpha knockout mouse model, an ASD mouse model displaying increased aggression during reciprocal social interaction [71,72]. This exaggerated response of male PZD mice toward social stimuli was also confirmed during an olfactory habituation/dishabituation test. Therefore, increased aggression likely leads to impaired social behavior in male PZD mice. In line with this, in reciprocal social interactions, PZD mice spent significantly more time in “resident behind intruder” events and displayed reduced oral-oral contact events, which also hints at increased aggression. Female PZD mice, in turn, that display less aggressive behavior, showed a significant impairment in the test for social novelty in the three-chamber box [53]. In addition, female PZD mice show impaired maternal social behavior towards their pups [54] (Table 1).

Repetitive behavior is a phenotype frequently displayed in ASD. Increased repetitive behavior has been reported in ASD mouse models such as Shank2 and Shank3 knockout (KO) mice [63,64,66,67,73]. Although stereotype self-grooming was not affected in PZD mice, marble-burying behavior was abnormal in PZD female mice [53]. In addition, PZD mice show striatal abnormalities. Striatal defects have been associated with repetitive behaviors [74]. In line with this, PZD mice show altered turning and circling behavior [36].

Additionally to the core features of ASD, maternal zinc deprivation induces an anxiety phenotype in the offspring, which was seen in the open field test and the paradigm of the elevated plus-maze [53]. Anxiety is an associated symptom of autism [75,76] and a phenotype detected in other ASD mouse models such as *Shank1*, *Shank2*, and *Shank3* KO mice [61,62,63,64,66,67,73] (Table 1).

Further, impaired nest building is frequently reported in ASD mouse models [71,77,78,79] and is also present in PZD mice [53]. Impaired nest building may hint at social and or cognitive deficits. Impaired motor learning may confirm a decline in cognitive abilities. Indeed, impairment in motor learning in PZD mice was seen in the rotarod test. This phenotype has also been reported in genetic ASD mouse models such as *Shank3* KO mice [66,67]. Also, impairment in learning and memory formation is one of the hallmarks of prenatal zinc deficiency reported in the literature [80,81,82,83,84].

Biochemical data support the idea that in vivo zinc deprivation affects SHANK protein dynamics within the PSD. This process leads to further downstream alteration of PSD composition regarding synaptic proteins and receptors, also known to be dysregulated in *Shank* KO ASD mouse models. Therefore, it may not be surprising that PZD mice share a behavioral phenotype similar to genetic ASD mouse models, particularly *Shank* KO mice (Table 1).

**Table 1:** Behavioral assessment of PZD mice in comparison to other ASD mouse models. Δex4–9: ^B^ refers to data of the mouse model published in [65] and ^J^ refers to data published in [66].

Mouse Model	Shank1	Shank2	Shank2	Shank3	Shank3	Shank3	Shank3	Shank3	Shank3	Neurexin 1 Alpha	PZD
	Exons 14–15/PDZ	Exons 6–7/PDZ	Exon 7/PDZ	Exons 4–9/ANK repeat (Δex4–9 ^B^)	Exons 4–9/ANK repeat (Δex4–9 ^J^)	Exons 4–7/ANK repeat (Δex4–7)	Exons 13–16/PDZ (Δex13–16)	Exon 11/SH3 (Δex11)	Exon 21 (Δex21)		
**Reference**	[61,62,85]	[64]	[63]	[65,73]	[66]	[67]	[67]	[63]	[86]	[71]	[53,54]
**Social Behavior**	Reduced sniffing by males in female-male interaction, normal juvenile social interaction	impaired sociability, impaired pup retrieval, impaired nest building	normal sociability, impairments in social novelty, normal initiation of social contact but impairments in maintaining social contact	mild social impairments in reciprocal social interaction (juveniles), normal sociability, normal social novelty, reduced social sniffing in males (male-female interaction	reduced sociability decreased bidirectional social interactions	normal initiation of social interaction, impaired social novelty	decreased frequency of nose-to-nose contact, decreased anogenital sniffing, impaired social novelty	not analyzed	Reduced time sniffing inanimate object in three-chamber test, impairments in social novelty (no preference of novel social target in KO)	increased social approach, increased aggression, reduced nest building	increased social approach, aggression, reduced nest building, decreased time spent in oral-oral contact (females)
**Ultrasonic vocalizations**	reduced number of USV	reduced number of USV during male-female interaction, increased latency to call	no difference in adults during male-male interaction, reduced calls, and longer latency to call during male-female interaction	reduced number of USV (adult mice)	increased number of calls (males), decreased number of calls (females)	not analyzed	not analyzed	not analyzed	No differences in the number of calls or latency to emit the first call in males in free-roaming male-to-female interaction during estrous	not analyzed	reduced number of USVs increased latency to call, reduced number of overtones with harmonics, decreased sound pressure level, increased latency to call during male-male or female-female interaction
**Repetitive behavior**	no increased self-grooming	no increased self-grooming in the home cage but increased in a novel object recognition task, hyperactivity	increased self-grooming, hyperactivity	increased self-grooming, inflexibility in reversal learning Morris water maze	an increased head pokes in hole board test, increased self-grooming	no increase in self-grooming	increase in self-grooming	increase in self-grooming	no increased self-grooming at 9–18 weeks old, increased self-grooming at older age	no increase in self-grooming	impaired marble burying (females), no significant increase in self-grooming
**Anxiety**	partial increased	increased	increased	not determined	not determined	not determined	increased	increased	Avoidance of light in the dark/light task, no differences in elevated plus maze or open field test	increased	increased
**Learning and Memory**	enhanced spatial memory, impaired fear conditioning, reduced motor learning	impaired spatial memory, normal novel object recognition	normal working memory, normal novel object recognition memory	impaired novel object recognition memory, normal spatial memory in Morris Water Maze, normal fear conditioning	impaired short- and long-term memory, impaired spatial learning in Morris Water Maze	not determined	normal spatial learning in Morris Water Maze	not determined	Impaired spatial learning and memory	no impairments in spatial, working, or episodic memory, short term or long memory	normal working memory (trend reduction), impairments in motor learning (rotarod)

### 2.2. Extracerebral Pathologies of PZD Mice

A growing amount of research indicates that abnormalities in the gastrointestinal (GI) system during development play a part in ASD. Said GI pathology might serve as a contributing factor in the development of ASD in offspring. Several studies link behavioral difficulties in autistic children to GI abnormalities [87,88,89]. In-between 19–70% of individuals with ASD show at least one GI symptom [88,90,91]. Among these, problems linked to altered intestinal barrier function are frequently reported [92,93]. In addition, investigations of the gut flora in ASD revealed an abnormal microbiome resulting in alterations in species, species numbers, and differences at the phylum level as a potential contributor to GI dysfunction [94,95]. Interestingly, it has been found that GI problems correlate with the severity of ASD behavioral pathology [96,97,98].

In line with these studies linking ASD to GI disorders in humans, PZD mice show a GI pathology, including alterations in tight junction proteins and increased barrier permeability [99]. Mechanistic studies using 3D gut organoids revealed that PZD is the primary driver of altered GI morphology and triggers pro-inflammatory processes that may be exacerbated by bacterial components transitioning through the compromised intestinal barrier. This, in turn, may feed back to bacteria by creating an alternative host environment, ultimately resulting in changed microbiota composition [99].

Consistent with this model and patients’ data [94,100,101], a dysbiosis in major microbiota phyla was found in PZD mice fecal samples [99]. Both PZD mice and ASD patients exhibit a significant increase in Actinobacteria and Proteobacteria phyla levels, along with a decrease in Firmicutes levels. Investigation of bacterial composition at class level revealed a significant increase in Actinobacteria, Bacilli, Erysipelotrichi abundance, and Gammaproteobacteria levels in PZD mice, mirroring findings in autistic children [102].

**Figure 1:**
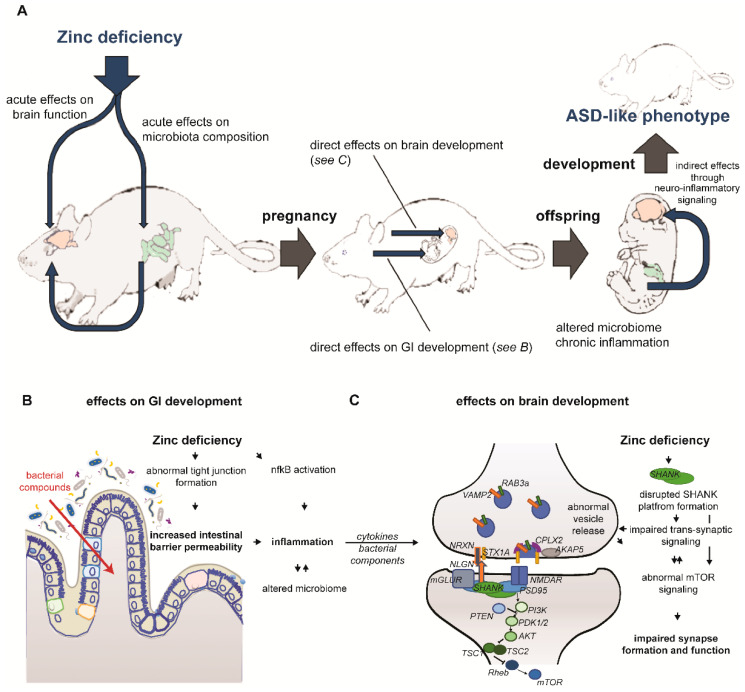
**Mechanisms involved in the development of the ASD-like phenotype in PZD mice.** (**A**) Acute zinc deficiency during pregnancy profoundly affects brain and intestinal organogenesis of the developing offspring, ultimately leading to direct effects on the brain and indirect effects through gut-brain interaction. The underlying mechanisms are as follows: (**B**) Zinc deficiency alters the expression and function of proteins critical for tight junction formation and activates immune signaling mediated by NFkB. As a consequence, intestinal barrier tightness is compromised and bacterial components can enter the circulation, thereby producing an immune response. This process fuels the already activated NFkB-driven pro-inflammatory signaling establishing a chronic inflammation. Leaky gut and inflammation create a host environment that is more suitable for specific bacteria, thus shifting microbiota composition significantly. Through released factors and/or bacterial components directly, the development of the brain is modified. (**C**) Additionally, zinc deficiency has direct effects on the brain, most notably on synaptogenesis and function. Zinc deficiency impairs SHANK dependent PSD scaffold formation, thereby affecting pre-synaptic function through trans-synaptic signaling via Neuroligin and Neurexin, which significantly affects synaptic vesicle function and trafficking by altering key proteins involved in the process such AKAP5. Abnormal vesicle release together with postsynaptic effects of abnormal SHANK physiology will alter neurotransmitter receptor signaling and ultimately affect important ASD-associated pathways such as mTOR signaling.

Impairments in GI integrity and a shift in intestinal microbiota composition and abundance in patients with ASD and PZD mice as well as other rodent ASD mouse models implicate a role of the GI system and its integrity in brain development and function via the microbiota-gut-brain axis. Several mechanisms for this have been proposed, including abnormal cytokine signaling caused by GI abnormalities. Due to challenges to the immune system (intestinal and systemic) derived from the gut bacterial community, alterations in inflammatory markers have been found in ASD patients and rodent models. In line with this, PZD mice show significant changes in the expression of inflammatory response markers [99]. Activation of NF-κB signaling by low zinc status in addition to further activation of changes in NF-κB signaling through toll-like receptors (TLR) responding to bacterial components gaining access through a leaky gut barrier results in altered expression levels of several CC chemokine ligands (CCLs) and interleukins in PZD mice. These data hint at a chronic systemic inflammation in PZD mice initiated by prenatal zinc deficiency and fueled by increased GI barrier permeability. Moreover, in PZD mice, changes in expression levels of interleukins in the brain suggest the presence of neuro-inflammatory processes [99]. While these findings in PZD mice link zinc deficiency, GI and immune system abnormalities to brain pathology and ASD behavior (Figure 1), a similar study in humans has not been conducted so far and highlights the need for multi-omics approaches in ASD [24].

### 2.3. CNS Phenotype of PZD Mice

Along with the behavioral alterations observed in PZD mice, morphological and molecular changes in the CNS induced by zinc deficiency during development and persisting into adulthood were observed. Zinc was repeatedly reported to be involved in processes of brain development, including neurogenesis, maturation, apoptosis, and synaptogenesis [103,104,105,106,107,108] and therefore insults like zinc deficiency during critical time windows of neuronal development can lead to permanent alterations in brain structure, connectivity, and functionality. Several studies showed that even mild zinc deficiency during prenatal development leads to long-term alterations in the brain affecting stem cell proliferation and diminished numbers of proliferating cells [104,107,109,110,111], lateralized expression of proteins [36] as well as synaptic function [54].

Mice that suffered from a mild zinc deficiency from gestational day 0–20 showed reduced brain zinc levels directly after birth that could be rapidly restored by an adequate zinc supply [54]. However, PZD mice show alterations in the brain structure of both the striatum and hippocampus [36,112]. An increase in the volume of both regions can be seen in PZD mice. This may correlate with some of the observed behavioral impairments. For example, striatum dysfunction is associated with stereotyped repetitive behaviors such as abnormal circling and altered marble-burying that has been reported in PZD mice [36,53]. Further, PZD mice demonstrate reduced motor learning behavior and nest building [53]. In other animal models for prenatal zinc deficiency, spatial learning impairment has been reported [26]. Memory and learning, in particular spatial learning, have been associated with the hippocampus. The morphological differences in the hippocampus of PZD mice were maintained into adulthood [112].

Intriguingly, a significant reduction in postsynaptic scaffold proteins of the SHANK family (SHANK1, SHANK2, SHANK3) and subunits of NMDA (N-methyl-D-aspartate) and AMPA (α-amino-3-hydroxy-5-methyl-4-isoxazolepropionic acid) receptors (GluA1, GluN1, GluN2B) was observed in synaptosomal-associated P2 fractions and fluorescently labeled cryosections. In addition, these animals showed lasting alterations in the composition of excitatory synapses, and the total number of synapses was slightly decreased [54]. Similar effects were reported in animals exposed to elevated levels of copper, leading to a secondary zinc deficiency during prenatal development due to the competing interaction of those trace metals. As in PZD mice, mice with prenatal copper overload show no changes in mRNA expression levels of Shank family members, but synapses were locally affected by a significant reduction in SHANK2 and SHANK3 as well as GluN1 proteins birth [113]. The reduction of PSD proteins induced by zinc depletion was often accompanied by an increase of the same proteins in the cytosolic fraction [54,105], arguing for effects on protein localization rather than translation or degradation.

Proteins of the SHANK (also known as Proline-rich synapse-associated protein ProSAP) family are major autism candidate genes [114]. They are major scaffold proteins within the PSD of excitatory synapses [115], and SHANK2 and SHANK3 are targeted to synapses via their zinc-binding C-terminal SAM domain [116,117,118]. Thus, zinc deficiency as an environmental factor for ASD is linked with the synaptic pathway identified from candidate genes studies on the level of SHANK protein regulation [33].

In contrast to SHANK2, SHANK3, and GluN2B protein levels that could be restored after birth by cross-fostering of PZD pups by mothers on a control diet during weaning, SHANK1, GluA1, and GluN1 protein levels remained significantly decreased [54]. However, even the transient loss of SHANK2 and SHANK3 during a critical window in brain development may significantly affect the establishment of brain connectivity that is relatively fixed after this period.

The loss of synaptic proteins by prenatal zinc restriction does not result in a general loss of all synaptic proteins but seems to change protein composition rather individually as levels of other PSD proteins such as PSD95, (guanylate kinase-associated protein) GKAP, and HOMER1, as well as receptor subunits like GluN2B or GluA2, were not affected. It is possible that a different yet stable scaffold forms at synapses in the absence of sufficient levels of SHANK2 and SHANK3 that seems to lack, in particular, the zinc-independent SHANK1 that may be dependent on a preformed SHANK2/3 platform at the PSD. If this model is correct, especially proteins exclusively interacting with SHANK1 may be depleted from synapses (Figure 1).

Zinc from both pre- and postsynaptic pools plays a crucial role in the formation, maturation, and persistence of the PSD with a major contribution of MT3 bound postsynaptic zinc released after synaptic activity [46,88]. As reported in mice, also in primary cultures, zinc depletion through chelation with TPEN or CaEDTA led to a reduction in SHANK2 and SHANK3 protein levels at synapses [54,105] that was accompanied by a decrease in PSD depth and area [105]. An increase in local zinc levels, i.e., after synapse activity, strengthens the PSD [105]. In line with this, SHANK3 labeling intensity at the PSD increases [34,119] It was shown that the levels of synaptic SHANK3 are crucial for the regulation of several ASD-associated proteins at excitatory synapses [120,121]. For example, SHANK3 interacts with the cytoplasmic tail of Neuroligins to coordinate trans-synaptic signaling through the Neurexin-Neuroligin complexes in rat hippocampal neurons. Thereby, synaptic levels of SHANK3 regulate AMPA and NMDA receptor-induced changes in the levels of presynaptic and postsynaptic proteins via Neurexin-Neuroligin signaling [119,120].

Disruption of the zinc-sensitive signaling system has been observed for Shank3 mutations related to ASD [121]. Intriguingly, an increase in dietary zinc levels reversed ASD-related behaviors in young *Shank3* KO mice and offspring of *Shank3* KO mice fed with a zinc-supplemented diet [122,123]. Whether the effects of dietary zinc supplementation result from direct zinc activity in the CNS or from restoring GI physiology is still unknown.

Intriguingly, zinc deficiency seems to result in more noticeable effects at excitatory synapses, possibly due to the physiological role of zinc/glutamate at zincergic synapses and the presence of SHANK proteins that are mostly limited to excitatory PSDs. In line with this, a reduction in excitatory synapse density but not in the number of inhibitory synapses was observed under zinc-deficient conditions in the striatum and cortex [54]. It is thus possible that the balance between excitation (E) and inhibition (I) is disturbed in these brain regions. E/I dysregulation through altered glutamatergic and GABAergic neurotransmission has long been suggested as an underlying factor in ASD [124]. Initially, it was hypothesized that an increase in the E/I ratio occurs, leading to hyper-excitability of cortical circuits. However, other studies have suggested that some ASDs are linked to a reduction in the E/I ratio [125,126]. In line with this, reports show that zinc and SHANK2 and SHANK3 regulate the biophysical properties of developing glutamatergic synapses through AMPAR [119], and the loss of SHANK2/3 proteins has mostly been associated with impaired glutamatergic signaling. However, several factors regulate neuronal excitability, such as intrinsic neuronal excitability, synaptic transmission, and homeostatic synaptic plasticity, and E/I is balanced by crosstalk within and between brain regions. A detailed electrophysiological characterization of the neuronal activity of PZD mice and their neuronal networks is so far missing, and the assessment of synapse loss did not analyze brain sub-regions or specific cell types. For example, a more detailed analysis of *Shank3* KO mice (*Shank3*^Δ9^ mice) revealed reduced excitatory transmission at Schaffer collateral synapses and increased frequency of spontaneous inhibitory synaptic events in pyramidal neurons. However, prelimbic layer 2/3 pyramidal neurons in the medial prefrontal cortex showed a decreased frequency of spontaneous inhibitory synaptic events [127]. In future, a more detailed analysis of neuronal activity and activation of neuronal networks of PZD mice is necessary to conclude whether the E/I ratio is increased or decreased, and in what brain regions.

Interestingly, synaptic mGluR5 proteins were upregulated in PZD pups that were nursed by control diet-fed dams [54]. In addition, cell culture experiments have shown that zinc is crucial for a HiK^+^ induced upregulation of mGluR5 [54]. So far, the mechanism behind this effect of zinc on mGluR5 levels is not known. Both increased and reduced mGlu5 functioning has been associated with ASD. In particular, dysregulation of mGlu5 signaling and thereby abnormal synaptic protein synthesis has been proposed as a critical factor besides abnormal mGlu5 receptor function due to interactions with its scaffolding proteins [128,129,130] (Table 2).

## 3. Conclusions

### 3.1. A Link between Genetic and Non-Genetic Factors in ASD through Zinc Signaling?

Most knowledge about the molecular mechanisms of ASD is based on KO mouse models. For example, mice with a mutation in FMR1 (Fragile X messenger ribonucleoprotein 1), TSC1,2 (TSC Complex Subunit 1), SHANK family members, Neurexin (NRXN), Neuroligin (NLGN), PTEN or SCN1A (Sodium voltage-gated channel alpha subunit 1) [132] have identified two significant pathways dysregulated in ASD, the mTOR/PI3K pathway and the NRXN-NLGN-SHANK pathway [133]. However, both of these pathways are closely linked and may be part of a superordinated NRXN-NLGN-SHANK-mTOR signaling network. Intriguingly, a subset of “environmentally responsive” genes tends to fall in this pathway. At synapses in the CNS, for example, we have previously shown that SHANK2 and SHANK3 are regulated by zinc at developing synapses [54,105] and lost from synapses in offspring of mice born from mothers with zinc deficiency [54]. Thus, we have shown that an environmental risk factor for ASD has been directly linked to the NRXN-NLGN-SHANK-mTOR pathway.

This pathway is key to regulating many processes in neurons, such as presynaptic vesicle dynamics [134,135], among others. Further, zinc deficiency is linked to gastrointestinal abnormalities such as alterations in the tight junction proteins and increases inflammation that may indirectly affect brain development in parallel. However, “synaptic” ASD-associated proteins have been found in the GI system as well [136,137], leaving the possibility that interaction between zinc and ASD-related genes also occurs in this tissue.

In a mouse model for ASD generated by prenatal exposure to lipopolysaccharide (LPS), which mimics infections by Gram-negative bacteria, prenatal zinc supplementation prevented communication impairments and social and cognitive ASD-like behaviors [138], linking two environmental factors for ASD. Thus, zinc deficiency and prenatal infection may act via the same pathomechanism. Interestingly, a drop in plasma zinc levels is seen after infection [139], which could temporarily deprive the embryo of its zinc supply and cause a release of inflammatory cytokines such as IL-6.

It was recently shown that an increase in the pro-inflammatory cytokine S100B during pregnancy affects the synaptic SHANK2 and SHANK3 levels in a zinc-dependent manner. Copy-number variations in *S100B* have been associated with ASD, and increased serum S100B has been found in ASD [50,140,141]. Animals exposed to high S100B levels in utero show reduced free zinc levels and SHANK2 in the brain. These mice displayed hyperactivity, increased stereotypic and abnormal social behaviors, and cognitive impairment on the behavioral level. Thus, likely, pro-inflammatory processes and abnormal zinc-signaling converge, ultimately leading to shankopathy or other synaptic deficits [131].

One can speculate that further environmental factors discussed in ASD can be associated with zinc deficiency [10]. For example, in humans, maternal diabetes increases the risk of developing ASD in the offspring [142,143]. The molecular mechanisms are currently not well understood. A key factor may be the relationship between diabetes, insulin, and zinc, which is complex with no apparent cause and effect relationships. Diabetes affects zinc homeostasis in many ways, and zinc plays a definite role in the synthesis, storage, and secretion of insulin. Moreover, it has long been speculated that toxic metals such as lead, or mercury play a role in the etiology of ASD [17]. As divalent metals, they may compete with zinc for absorption, thereby lowering the bioavailability of zinc.

**Figure 2:**
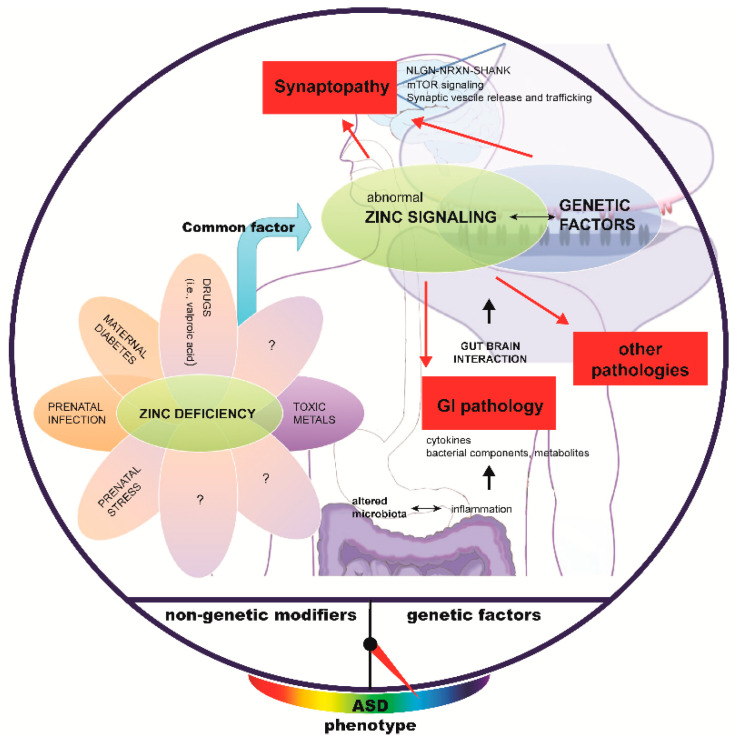
**Associations between risk factors for autism.** Evidence is mounting that several risk factors for ASD, such as maternal diabetes, prenatal stress, the use of certain drugs, the presence of toxic metals, and prenatal infection, all previously considered independent factors, converge on abnormal zinc signaling on biological level. Abnormal zinc signaling affects the development of the brain and GI system, and likely, other organ systems. Notably, zinc signaling is key to processes defined by genetic factors linked to ASD. Thus, abnormal zinc signaling is able to trigger and modify ASD pathology caused by a variety of genetic factors.

Intriguingly, it was shown that in two other non-genetic animal models for ASD, one modeling prenatal infection by bacterial lipopolysaccharide (LPS) injection, and one by prenatal exposure to valproic acid (VPA), ASD-like behavior is rescued in mice by treatment with zinc [138,144] suggesting a link between these treatments and zinc homeostasis. Intriguingly, PZD and VPA models also share alterations in microbiota composition. Higher *Alphaproteobacteria* class-level-abundance in PZD mice and a decrease in *Clostridia* on class level [99] correspond to microbiota abundance level in a VPA rat model of ASD [145]. Additionally, low abundance on the class level in *Deltaproteobacteria* in PZD aligns with microbiome data from a murine VPA model of ASD [146]. Thus, it may be possible that zinc deficiency is at the base of a large share of ASD cases (Figure 2).

### 3.2. Future Perspectives—Prevention and Treatment Strategies

Given the tight relationship between ASD-like behavior and prenatal zinc deficiency in mice, and the association of a high incidence of low zinc levels with ASD in humans, several questions emerge. Two of the central questions are whether low zinc levels are likely to occur during pregnancy in humans and what possible causes for this may be.

There are two main pools of zinc within the body: a slowly zinc exchanging pool and a pool that rapidly exchanges zinc with the plasma. Although the latter contains only 10% of the body’s zinc, it is the one that is primarily reactive to the amount of zinc absorbed from dietary sources. It is also the first to be depleted under conditions of zinc deficiency and the source of the embryo’s zinc supply [77]. During pregnancy, there is increased demand for zinc. While the metabolic zinc requirement of 2.5 mg/d for an adult woman is generally met when consuming daily 10 to 15 mg zinc, an additional 5–10 mg zinc per day must be consumed during pregnancy. Further, during lactation, the daily requirement increases by another 2.5 mg per day [118].

Unfortunately, there is limited data available on whether the required daily intake is met in pregnant women. However, it has been estimated that pregnant women could struggle to meet the required daily intake even in industrialized nations. In some studies, it has been estimated that even up to 80% of pregnant women are zinc deficient [147,148], although regulation of zinc homeostasis on the cellular level in pregnant women may overcome deficiencies in zinc intake to a certain extent [149].

Although zinc may be present in foods, the bioavailability of zinc can be affected by several factors such as drugs, nutritional supplements, and other food components. In a western mixed diet, the bioavailability of zinc is about 20–30% [150,151]. This bioavailability can, for example, further be reduced through the presence of high levels of phytates such as inositol hexaphosphates and pentaphosphates in the diet [152]. Since phytic acids are highly present in a vegetarian diet resulting in a low bioavailability of zinc that additionally is higher concentrated in meat, a recent study investigated whether pregnant vegetarian women meet their required daily intake of zinc. However, neither vegetarian nor non-vegetarian groups met the recommended dietary allowance for zinc [153].

More importantly, high levels of Ca and Fe, and folic acid, commonly prescribed and supplied at higher levels during pregnancy, have been shown to decrease zinc absorption [154,155,156,157]. Folic acid has been reported to increase fecal Zn excretion, indicating an inhibitory effect on Zn absorption [158,159]. Intriguingly, recent studies suggest that excessive amounts of folate might increase the risk for the occurrence of ASD [160]. One might speculate that the underlying pathomechanism could be a secondary prenatal zinc deficiency.

To investigate the antagonistic effects, in a recent study, mice were fed a special diet containing either adequate zinc concentrations, inadequate zinc concentrations, or adequate zinc concentrations in the presence of phytic acid, Ca/Fe, and folic acid [161,162]. While control animals displayed normal Zn levels in whole blood and, as expected, mice on a zinc-deficient diet showed a significant reduction of zinc levels, a zinc deficiency similar to animals fed a zinc-deficient diet was induced in mice on the control diet but with increased levels of zinc uptake antagonists [161,162].

Therefore, mineral and vitamin supplementation during pregnancy should consider interaction with other dietary factors. Supplements should carefully balance the levels of trace metals, as homeostasis between different trace elements is tightly connected [163]. Unfortunately, to our knowledge, no study so far followed dietary zinc uptake or measured zinc levels in pregnant women and followed their offspring for several years until a diagnosis of ASD could be reliably made. Besides, to our knowledge, no study investigating the effect of maternal zinc supplementation on incidence rates of ASD has been published so far. A factor that might explain the lack of knowledge about zinc levels during pregnancy is the difficulty in accurately determining zinc concentrations in daily clinical practice.

There are no good indicators or biomarkers to assess zinc status available at present. Subclinical zinc deficiency usually does not lead to symptoms that expressly point to zinc deficiency. Although there have been 32 biomarkers for zinc status proposed [164], only 3 out of 32 biomarkers may be helpful (plasma, urine, and hair zinc concentrations) [165]. However, none of the methods currently used can be recommended because each has its limitations [166]. For example, plasma zinc levels fluctuate naturally because of a pregnancy.

**Figure 3:**
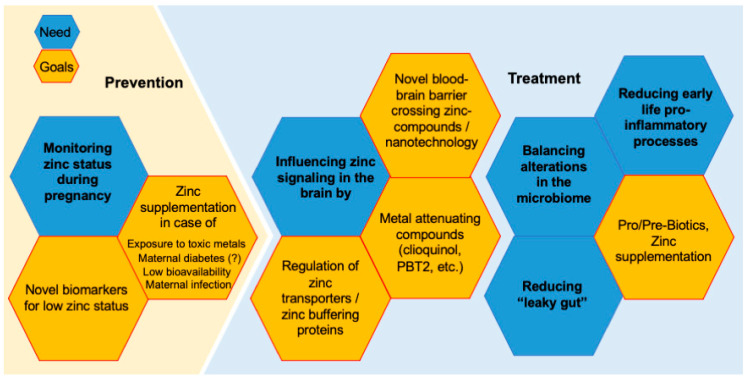
**Prevention and treatment approaches based on data from PZD mice.** Balancing trace metal levels, especially preventing zinc deficiency during pregnancy may be a promising prevention strategy. However, the bioavailability of zinc needs to be considered that can be impacted by folic acid supplementation and mineral supplements with high levels of Fe, Ca, and Cu. The exposure to toxic metals needs to be considered. Given that maternal infection and maternal diabetes are tightly linked to trace metal alterations, if infection or diabetes occurs, zinc supplementation may be an important preventive approach. In contrast, zinc supplementation may have a limited effect on patients with ASD after the critical time window of brain development in utero. Nevertheless, influencing zinc signaling locally in the brain and synapses may be a promising target for drug development. To that end, new zinc delivering compounds (zinc amino-acid conjugates, zinc ionophores, etc.) and nanoparticles, and drugs targeting proteins (MTs (metallothioneins), zinc transporters) regulating zinc homeostasis can be developed. Balancing alterations in the microbiome needs to be explored in terms of the effects on zinc availability. Finally, chronic inflammation resulting from the GI pathology linked to zinc deficiency could be addressed as early as possible as an intervention in ASD.

However, zinc supplementation may be a measure to decrease the risk of ASD and should be closer investigated as prevention in the future. Although zinc supplementation is considered not toxic even at higher doses as the required daily intake, further studies regarding safety during pregnancy are warranted. Further, new supplements such as zinc-amino acid conjugates (ZnAA) that are not affected by zinc uptake antagonists such as phytic acid or folic acid [131] should be developed to increase the bioavailability of zinc during pregnancy [167] (Figure 3).

Treatment of GI problems was suggested to be associated with improved behavior [88,168,169,170]. However, little is known how these treatments, like pre- or probiotics, will affect zinc absorption in the intestines. Given that zinc is an essential trace metal for bacteria, but different species have different demands, there is a close but so far unexplored link between the microbiota composition and the metallome of the body [24] (Figure 3).

As discussed above, zinc deficiency during development disrupts the synaptic activity-dependent increase of SHANK2 and SHANK3 and influences NMDA receptor signaling [121,171,172,173]. Thus, it may be possible that the modulating ability of zinc may be an interesting feature that influences the ASD associated pathway at synapses. Indeed, trans-synaptic mobilization of zinc induced by using the zinc-ionophore clioquinol has been shown recently to rescue social deficits in two different mouse models of ASD [174].

Based on these results, control of local zinc concentrations at synapses through zinc transporters may be a promising approach, and synaptic zinc transporters an interesting drug target. Further, metal protein attenuating compounds (MPACs) that have been proposed for the treatment of cognitive impairment due to Alzheimer’s dementia [141,175] may show beneficial effects in ASD. Unfortunately, to our knowledge, these drugs have not been investigated in ASD mouse models or human patients so far, although MPACs such as PBT2 have been proven safe and tolerable in humans in clinical trials (Figure 3).

Thus, taken together, based on the results using mouse models with trace metal imbalances such as early life zinc deficiency but also copper overload, it is possible that a group of ASD cases may be regarded as disorders of trace metal metabolism. This, so far, new and underrated fact may open new possibilities for research in ASD and the development of novel treatment strategies.

## Figures and Tables

**Table 2:** Overview of the synaptic phenotype of zinc deficiency regarding protein composition during brain development and in vitro: * brain region-specific; ** changes only observed in the presence of Shank3; ^†^ reported alterations in synapse density; ^#^ normalized after zinc repletion; ZnD: Zinc deficient; IHC: Immunohistochemistry; MS: mass spectrometry; WB: Western Blot; ICC: Immunocytochemistry; DIV: day in vitro; PD: postnatal day.

Protein	Effect	Induction of ZnD by	Method of Detection	Model System		Reference
				In Vitro	In Vivo	
SHANK2	reduced	TPEN, CaEDTA, High S100B	ICC, WB	x		[54,105,131]
	reduced (PD3) ^#^	PZD, Prenatal S100B	IHC, WB		x	[54,131]
SHANK3	reduced	TPEN, CaEDTA, High S100B	ICC, WB	x		[54,105,131]
	reduced (PD3) ^#^	PZD	IHC, WB		x	[54] ^†^
SHANK1	reduced	CaEDTA	ICC	x		[54] ^†^
	reduced (PD3)	PZD	IHC, WB		x	[54] ^†^
HOMER1b/c	reduced	TPEN, CaEDTA	ICC, WB	x		[105] [54] ^†^ [121] **
	reduced (PD3) ^#^	PZD	WB		x	[54] ^†^
VGLUT1	reduced	TPEN	ICC	x		[121] **
mGluR5	blocked increase after HiK+ stimulation	TPEN, CaEDTA	ICC, WB	x		[54]
	reduced (PD3)	PZD	WB	x		[54]
GluA1	reduced (PD3)	PZD	WB		x	[54]
GluN1	reduced only in Shank1 deficient synapses	TPEN	ICC	x		[105]
	reduced (PD3)	PZD	WB		x	[54]
GluN2B	reduced (PD3) ^#^	PZD	WB		x	[54]
PSD 95	reduced only in Shank1 deficient synapses	TPEN	ICC	x		[105]
	no change	TPEN, CaEDTA	ICC, WB			[54]
	no change	PZD	WB		x	[54]
GluN2A	reduced (PD3)	PZD	WB		x	[54]
GluA3 *	reduced (PD3) ^#^	PZD	WB		x	[54]
GluA2 *	reduced (PD3) ^#^	PZD	WB		x	[54]
GKAP *	reduced (PD3) ^#^	PZD	WB		x	[54]
ARRB2 *	loss of lateralized expression	PZD	qRT-PCR, WB *		x	[36]
FEZ1 *	loss of lateralized expression	PZD	qRT-PCR, WB *		x	[105]

## Data Availability

Not applicable.
